# Another Tragic Pandemic Strikes: It Is Suicide

**DOI:** 10.1192/j.eurpsy.2023.2359

**Published:** 2023-07-19

**Authors:** J. Petta, A. L. Falcão, G. Soares, A. Lourenço

**Affiliations:** Centro Hospitalar Psiquiátrico de Lisboa, Lisboa, Portugal

## Abstract

**Introduction:**

Another pandemic besides COVID-19 stalks the land. This one takes a heavy toll on the young.

Suicide occurs throughout the lifespan and is the second leading cause of death among 15-29 years old globally.

**Objectives:**

The objective of this review is to highlight for another tragic pandemic, with main emphasis on the preventable character.

**Methods:**

Data was obtained through an internet-based literature review, using the research platform Pubmed and the World Health Organization website. Seven articles from the last two years were included.

**Results:**

Improved surveillance and monitoring of suicide attempts and self-harm is a core element of suicide prevention and desirable worldwide.

A public health surveillance system based on medical records would provide and disseminate data that would guide and prioritize the best interventions in each context and contribute to an effective overall suicide prevention strategy.

**Conclusions:**

Close to 800 000 people die due to suicide every year, which is one person every 40 seconds.

According to current data, for each adult who died by suicide there may have been more than 20 others attempting suicide.

Effective and evidence-based interventions can be implemented to prevent suicide and suicide attempts.

**Disclosure of Interest:**

None Declared

